# Effect of Emamectin Benzoate on Mortality, Proboscis Extension, Gustation and Reproduction of the Corn Earworm, *Helicoverpa zea*

**DOI:** 10.1673/031.010.8901

**Published:** 2010-07-02

**Authors:** Juan D. López, M. A. Latheef, W. C. Hoffmann

**Affiliations:** USDA, ARS, SPA, SPARC, Areawide Pest Management Research Unit, College Station, TX

**Keywords:** emamectin benzoate, *Helicoverpa zea*, feeding, adult control, attracticide, larval hatch, mating, pupation

## Abstract

Newly emerged corn earworm adults, *Helicoverpa zea* (Boddie) (Lepidoptera: Noctuidae) require a carbohydrate source from plant or other exudates and nectars for dispersal and reproduction. Adults actively seek and forage at feeding sites upon eclosion in the habitat of the larval host plant or during dispersal to, or colonization of, a suitable reproductive habitat. This nocturnal behavior of *H. zea* has potential for exploitation as a pest management strategy for suppression using an adult feeding approach. This approach entails the use of a feeding attractant and stimulant in combination with a toxicant that when ingested by the adult will either reduce fecundity/fertility at sub-lethal dosages or kill the adult. The intent of this study was to assess reproductive inhibition and toxicity of emamectin benzoate on *H. zea* when ingested by the adults when mixed in ppm active ingredient (wt:vol) with 2.5 M sucrose as a feeding stimulant. Because the mixture has to be ingested to function, the effect of emamectin benzoate was also evaluated at sub-lethal and lethal concentrations on proboscis extension and gustatory response of *H. zea* in the laboratory. Feral males captured in sex pheromone-baited traps in the field were used for toxicity evaluations because they were readily available and were more representative of the field populations than laboratory-reared adults. Laboratory-reared female moths were used for reproduction effects because it is very difficult to collect newly emerged feral females from the field. Emamectin benzoate was highly toxic to feral *H. zea* males with LC_50_ values (95% CL) being 0.718 (0.532–0.878), 0.525 (0.316–0.751), and 0.182 (0.06–0.294) ppm for 24, 48 and 72 h responses, respectively. Sub-lethal concentrations of emamectin benzoate did not significantly reduce proboscis extension response of feral males and gustatory response of female *H. zea.* Sublethal concentrations of emamectin benzoate significantly reduced percent larval hatch of eggs and mating frequency of female *H. zea.* Larval survival to the pupal stage was also significantly reduced by ingestion of emamectin benzoate by female *H. zea.* These data suggest that emamectin benzoate is a useful toxicant in an attract-and-kill control strategy against *H. zea.* Field studies are warranted to validate the results reported in this study.

## Introduction

The concept of using feeding attractants and stimulants in combination with a toxicant for adult control of noctuid pest has been documented (Lopez et al. 2000). The feeding attractants would be used to attract adults to specific areas treated with a feeding stimulant mixed with a toxicant where the adults would be induced to feed. The ingested toxicant would be expected to prevent/reduce reproduction at sub-lethal concentrations or kill the adults at lethal concentrations. A number of feeding attractants have been evaluated/identified for both sexes of several noctuid pest species (Pair and Horvat 1997; López et al. 2000; De Camelo 2006; Meagher and Landolt 2008).

The corn earworm, *Helicoverpa zea* (Boddie) (Lepidoptera: Noctuidae), and its closely related species, the tobacco budworm, *Heliothis virescens*, occur throughout the temperate and tropical regions in the Americas (Bergvinson 2005). The estimated annual cost of both species on all crops in the USA is estimated to be $1 billion irrespective of an expenditure of $250 million on insecticide application to control these pests (Fitt 1989). The preferred host of *H. zea* is corn, *Zea mays* (Quaintance and Brues 1905, Lincoln and Isley 1947, Hardwick 1965). In the Brazos Valley of Texas, field corn serves as a “nursery” crop and produces large populations of *H. zea* adults that move into cotton in late June to early July and establish themselves as one of the important pests of that crop (López 1976). In corn, most eggs are deposited on fresh silk tissue of developing ears. The hatchlings travel down the silk channel and begin feeding on kernels within the ear tip. Larvae are covered by husk tissue and are protected from insecticidal contact.

Even sweet corn hybrids expressing *Bacillus thuringiensis* toxin do require some insecticidal applications to produce fresh market crop (Burkness *et al.* 2001; Speese III *et al.* 2005).

Emamectin benzoate belongs to the avermectin group of chemicals produced by the soil-dwelling actinomycete (NRRL 8165) alias, *Streptomyces avermitilis* (Burg *et al.* 1979). It possesses excellent insecticidal potency against neonates of *H. zea* in foliar application with an LC_90_ value of 0.002 µg/ml (White *et al.* 1997). Argentine *et al.* (2002) found that the LC_90_ values for emamectin benzozte ranged from 0.0050 to 0.0218 µg/ml for six species of Lepidoptera. Dunbar *et al.* (1998) reported that emamectin benzoate was very effective in controlling *H. virescens* and *H. zea* larvae at low active ingredient rates (0.0084–0.084 kg /ha). Jansson and Dybas (1996) reported that emamectin benzoate is stored as a reservoir in plant parenchyma tissues and this accounts for its long residual activity against several phytophagous insects. Jansson *et al.* (1996) reported that solid formulations of emamectin benzoate were as efficacious as the emulsifiable concentrate formulations in controlling *H. virescens* and beet armyworm, *Spodoptera exigua* (Hübner) larvae.

Joyce (1982) estimated that *H. zea* adults are 10 to 100 times more susceptible than larvae to insecticides. Leona and Slynko (1998) reported that higher detoxification associated with cuticular penetration, internal accumulation, excretion of applied toxicants and their metabolites occurred more rapidly in larvae compared with that in adults. In spite of reported advantages of targeting adult *H. zea* rather than larvae, no study has been reported on toxicity of emamectin benzoate, relative to feeding response, mortality, and reproduction for the adult insect.

An appreciation of nocturnal activity of *H. zea* has led to the belief that “attract-and-kill” strategy targeting highly mobile adult populations of the insect at their eclosion sites before their dispersal to other host plants for reproductive functions may likely be potentially efficacious against this insect. Lingren *et al.* (1988) studied the nocturnal and post-emergence behavior of *H. zea* and found that newly-emerged adults sought food as their first nightly activity as soon as they were able to fly. Lingren *et al.* (1990) also demonstrated that upon emergence, *H. zea* fed on thiodiocarb-baited sorghum-water mixture, that was banded around corn stubble, caused major mortality of the insect. Beerwinkle *et al.* (1993) found large populations of virgin *H. zea* feeding at <1 h after sunset on honey dew exudates of ergot, *Claviceps paspali* (F. L. Stevens & J. G. Hall) growing near their emergence habitat.

A major drawback to the development of “attract-and-kill” strategy is the lack of fundamental information vis-à-vis toxicants and their effects on the target insect. In this study, the effect of emamectin benzoate against *H. zea* was examined relative to toxicity, proboscis extension, gustatory response, reproduction, and survival of the progeny when it was provided in a feeding stimulant solution to the adult insect.

## Materials and Methods

### Test insects

*Feral males.* Natural populations of males were chosen to study lethal concentration, proboscis extension response and mean lethal time because feral males are readily available throughout the growing season through captures in pheromone-baited traps. Pheromone traps were established using 75–50 Texas wire cone sex pheromone traps (Hartstack *et al.* 1979, Hartstack and Witz 1981) baited with laminated plastic Zealure (Luretape® Insect Attractant Dispenser, Hercon Environmental, www.herconenviron.com). The wire cone measured 75 cm in diameter reduced to a 50 cm opening for moth's entry at the bottom of the cone. The traps were established in close proximity to cotton fields in the Brazos River Valley near College Station, TX, and were serviced every morning. Before the tests were conducted, males captured in the morning were fed deionized water through water-soaked sanitary pads.

*Laboratory-reared moths. H. zeas* were reared in the laboratory from eggs obtained from the Southern Insect Management Laboratory, USDA-ARS, Stoneville, MS., using similar techniques described previously (López and Lingren 1994). Larvae were reared on soybean-wheat germ diet (V 0600, Stonefly Heliothis Diet, Ward's Natural Science, www.wardsci.com). Approximately 4 g of the diet was dispensed into a plastic soufflé cup using a caulking gun and an individual larva was placed on the diet and sealed with a lid. About 3 weeks thereafter, pupae were harvested, sorted by sex, and male and female pupae were placed separately in 3.78 L jars for moth emergence. All rearing and testing with lab oratory-reared moths were conducted during the day in a laboratory maintained at 23.9 ± 0.38°C, RH 64.5 ± 4.6 % and a photoperiod of 14:10 L:D.

### Test solutions

A 1000 ppm stock solution of emamectin benzoate (MK-0244 5% SG) supplied by Merck Research Laboratories, (Syngenta AG, www.syngentacropprotection.com) was prepared in deionized water. Serial dilutions ranging from 0.0125 to 200 ppm were then prepared by dilution with 2.5 M sucrose solution (grade II, Sigma Chemical Co., www.sigmaaldrich.com). All tests were compared with 2.5 M sucrose solution as the check. Test solutions were stored in a refrigerator, and were warmed to laboratory temperature before being used.

### Determination of lethal concentration

The lethal concentration values were determined to optimize toxicant concentration in an attracticide formulation. Feral males captured in pheromone-baited traps were fed using the feeding apparatus (described below) for 30 min on each toxicant concentration. Moths were fed with a range of concentrations of emamectin benzoate. Since feral males were not a limiting factor in the number of insects used per treatment, ten moths were fed on each concentration in each replication. Each concentration was replicated 5 times. A total of 300 moths were used in this test. Each moth was placed individually inside a onequart bottle, and was examined for mortality at 24, 48 and 72 h thereafter. A male was considered dead when it could not right itself when placed upside down.

### Determination of lethal time

To determine mean time to death, ten moths were fed emamectin benzoate at each concentration comprising of 1.3 ppm (1XLC_90_), 6.5 ppm (5XLC_90_), 13 ppm (10XLC_90_), 32.5 ppm (25XLC_90_) and 65 ppm (50XLC_90_). Each adult was placed individually in a sealed plastic soufflè cup after feeding and observed for mortality at 15, 30 and 45 min and every hour thereafter until a 6 h period when checking was increased to 12, 18, and 24 h. The mid-point of the interval during which the adults were considered dead was used in calculations.

### Determination of proboscis extension response

The ability to elicit proboscis extension is a pre-requisite for feeding and the determination of this response for use of emamectin benzoate as a toxicant in a feeding stimulant/attracticide formulation is critical for the development of attract-and-kill technology for *H. zea* control. The methods used to determine the proboscis extension response were similar to those described by López *et al.* (1995). Briefly, it comprised of holding each moth with the index finger and the thumb, the front tarsi were touched to the test solutions in a porcelain multi-well plate by pulling the front legs across it while minimizing contact of other body parts with the solution. Using a 2.5 M sucrose solution, the proboscis extension response was evaluated soon after sunset in an insectary under red light using feral males captured in pheromone-baited traps. Concentrations of emamectin benzoate from 4, 20, 40, 100 and 200 ppm were used to assess the proboscis extension response. Each concentration was replicated ten times with ten moths in each replication. If the proboscis was completely extended to contact the test solution, a positive response was recorded, and if no proboscis was extended, a negative response was recorded. Moths which elicited a partial response were removed from the tests. While this technique is different than those used for honeybees (Buckbee and Abramson 1997), which requires a training period, the authors have found this to be reliable and time-efficient method.

### Determination of gustatory response

**Figure 1.**
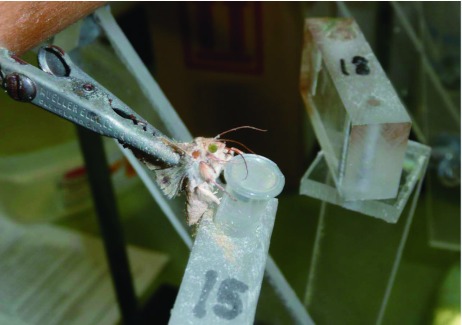
Feeding apparatus used to conduct gustatory response of *Helicoverpa zea.* See descriptions. High quality figures are available online.

The gustatory response of feral males and laboratory-reared females was determined using a feeding apparatus (Figure 1) (López and Lingren 1994; Clemens 1996). Moths were held in position with the wings folded behind the thorax and pinched together with the alligator clips. A polystyrene disposable centrifuge tube (0.5 ml) containing the test solution was placed in a hole drilled in a Plexiglas® block. Two piece hinged Plexiglas arms about 40 cm long were drilled, screwed, and glued to the dowels behind the alligator clips. The hinged arms were used to position the feeding tube in front of the proboscis of the moth, allowing the insect to feed from the tube, and avoiding contact with the moth's legs and thorax. If the moth did not extend its proboscis, it was teased with the tip of an insect pin to contact the test solution.

Only laboratory-reared females that emerged during the previous night and feral males obtained in the morning from pheromone-baited traps were used. To determine the gustatory response, moths were mounted individually in the feeding apparatus and were offered the test solutions contained in a disposable polystyrene centrifuge tube (0.5 ml). The amount fed was determined from the differences between the before and after feeding weights of the tubes, after correcting for evaporation loss from tubes kept aside as control in each concentration.

### Determination of fecundity and fertility

Each newly emerged laboratory-reared female was fed emamectin benzoate until satiation, which did not exceed 30 min, and was paired with an untreated male. The glass jars containing test moths were closed with a paper towel. A strip of paper towel was suspended from the mouth of each jar, which provided a substrate for the moths to climb and lay eggs. The moths were fed 10% sucrose solution in a 25 ml plastic soufflè cup with a lid through which a cotton wick was inserted. Dead males were replaced with live males during the test. Moths that remained in copula while mating were removed from the study.

Two tests with slightly different divisions of concentrations between 0 and 1 ppm were conducted to evaluate the reproductive effects of emamectin benzoate at sub-lethal concentrations. Females were fed emamectin benzoate at 0.0125, 0.025, 0.05, 0.075 and 0.1 ppm and 0.05, 0.1, 0.2, 0.6 and 1.0 ppm in Tests 1 and 2, respectively. Soon after gustation, the adults were transferred to clean jars, and paired with an untreated male. Beginning the 2^nd^ day, numbers of eggs in the used jars were counted and a sample of ca. 30 eggs was collected from the paper towel in each jar for three consecutive days from each concentration in each replication. Fecundity was assessed from 5 females in each concentration in each replication because of the difficulty in counting the eggs as they were deposited indiscriminately on paper towels, on the inside surface of the jars and on cotton wicks.

**Table 1.**
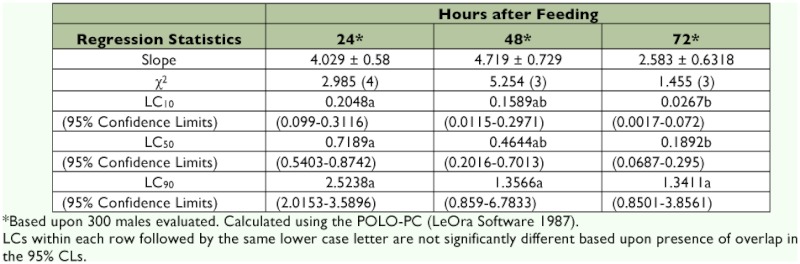
Lethal concentration (LC) data (± SEM) for pheromone trap-captured bollworm males fed emamectin benzoate mixed with 2.5 M sucrose.

**Table 2.**
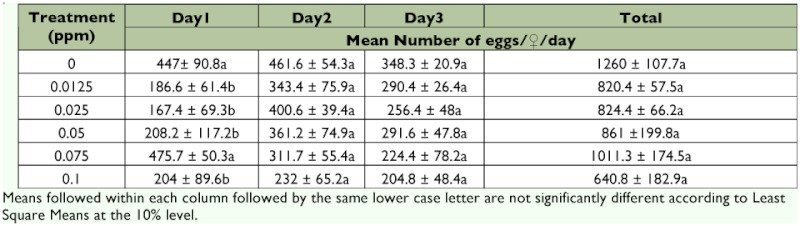
Number of eggs (± SEM) deposited by bollworm fed emamectin benzoate mixed with 2.5 M sucrose during a 3-day period, Test 1.

Eggs were set aside in 25 ml plastic soufflé cups sealed with a lid for determining larval hatch, which was checked for three consecutive days. Eggs from unmated females were excluded from determination of egg viability. At the end of each test, female moths were dissected under a 30X stereo zoom microscope to determine mating frequency by counting the number of spermatophores in the bursa copulatrix. The male *H. zea* moth transfers a spermatophore to the female at the time of mating (Callahan 1958).

During each egg viability check, a minimum of ten larvae were removed from the cups with the egg samples in each concentration in each of five replications during each of three consecutive days and reared individually to pupa on the insect diet. Approximately three weeks after the placement of larvae on the diet, each soufflè cup was examined for the presence of pupae. Pupae were sexed and counted.

### Data analyses

Analyses of variance of the data were conducted using PROC GLM procedures (SAS 1988). When F-values for treatment were significant at the 5% level, means were separated using the Least Square Means procedure. In the few instances where F-values were significant at the 10% level, alpha was set at 0.1 when means were separated. Lethal concentration values were determined using the POLO Software (LeOra Software 1987). Significant differences in lethal concentration values were determined based upon the lack of overlap in confidence limit values at the 95% level. The nonparametric PROC NPAR1WAY procedure was conducted to determine if ingestion of emamectin benzoate by *H. zea* females influenced survival of larvae to the pupal stage. Bar graphs of the raw data are shown with the ANOVA statistics computed by PROC NPAR1WAY procedure in lieu of Wilcoxon scores. The Kruskal-Wallis test (option = exact) is shown as well. The goodness of fit, χ^2^ statistic, was used as the test criterion to describe the distribution of pupae by gender at the 5% level of probability.

**Figure 2.**
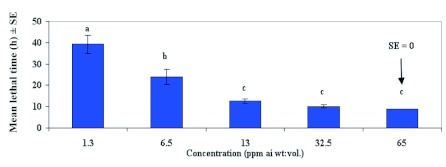
Mean lethal time (± SEM) in hours for feral male *Helicoverpa zea* fed emamectin benzoate mixed with 2.5 M sucrose solution. Means followed by the same lower case letter are not significantly different according to Least Square Means Procedure (P=0.05). High quality figures are available online.

## Results and Discussion

### Mortality

Table 1 shows that for feral males captured in pheromone-baited traps, the dosage mortality equation approximated the 24, 48 and 72 h responses (χ^2^ = 2.985 with 4 df for 24 h response; χ^2^ = 5.254 and 1.455 each with 3 df for 48 and 72 h responses, respectively). The χ^2^ values were less than the tabular values for appropriate degrees of freedom. The LC_50_ {(95% CL (confidence limits)} values for feral males were 0.7189 (0.5403–0.8742), 0.4644 (0.2016–0.7013), and 0.1892 (0.0687–0.295) ppm for 24, 48 and 72 h responses, respectively. The LC_50_ value for 24 h response was not significantly different from that for 48 h response. Also, the LC_50_ value for 48 h response was not significantly different from that for 72 h response. However, LC_50_ value for 24 h response was significantly different from that for 72 h response.

Mean lethal time for 48 h response varied significantly between concentrations of emamectin benzoate (F = 24.91; df = 4, 45; P<0.001). Figure 2 shows that the mean lethal time (LT) was 39.3 h at 1.3 ppm (1X LC_90_), 24 h at 6.5 ppm (5XLC_90_), 12.6 h at 13 ppm (10XLC_90_), 10.2 h at 32.5 ppm (25XLC_90_) and 9 h at 65 ppm (50XLC_90_). Relationship between LTs and concentrations of emamectin benzoate was inversely related and appears to level off asymptotically. The LTs at 13 ppm and higher were not significantly different and suggests an optimum concentration of 13 ppm for causing the quickest mortality.

### Proboscis extension response

**Figure 3.**
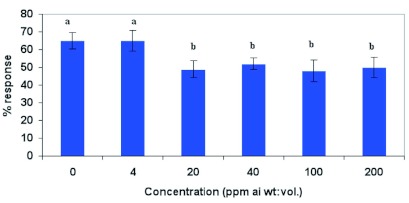
Mean proboscis extension response (± SEM) of feral male *Helicoverpa zea* to emamectin benzoate mixed with 2.5 M sucrose solution. Means followed by the same lower case letter are not significantly different according to Least Square Means Procedure (P=0.10). High quality figures are available online.

Emamectin benzoate significantly influenced proboscis extension response of feral males (F = 2.37; df = 5, 54; p<0.10). Proboscis extension response was significantly depressed at 20 ppm and above compared with males fed 2.5 M sucrose solutions. Emamectin benzoate, however, did not significantly depress proboscis extension response of males at 4 ppm (Figure 3). These results suggest that concentrations of emamectin benzoate below 4 ppm can be used as a toxicant under field conditions in conjunction with a feeding stimulant for control of *H. zea.*

### Gustation

**Figure 4.**
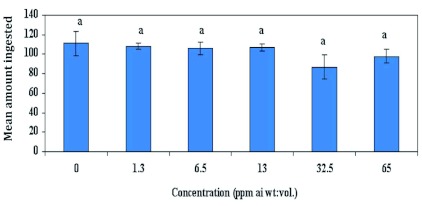
Gustatory response of feral male *Helicoverpa zea* fed emamectin benzoate mixed with 2.5 M sucrose. High quality figures are available online.

**Figure 5.**
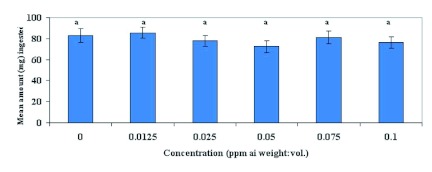
Mean gustatory response (± SEM) of laboratory-reared female *Helicoverpa zea* to emamectin benzoate mixed with 2.5 M sucrose solution in Test I. Means followed by the same lower case letter are not significantly different according to Least Square Means Procedure (P=0.05). High quality figures are available online.

**Figure 6.**
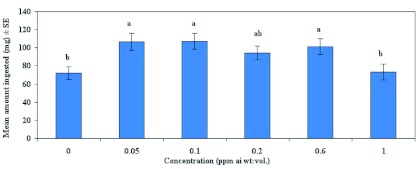
Mean gustatory response (± SEM) of laboratory-reared female *Helicoverpa zea* fed emamectin benzoate mixed with 2.5 M sucrose solution (Test 2). Means followed by the same lower case letter are not significantly different according to Least Square Means Procedure (P=0.05). High quality figures are available online.

Figure 4 shows that gustatory response of feral males at the concentrations of emamectin benzoate (1.3, 6.5, 13.0, 32.5 and 65 ppm) showed no significant difference between treatments when compared with males fed 2.5 M sucrose solutions (F = 1.12; df = 5, 54; p >0.05). Figure 5 shows that in Test 1 when laboratory-reared females were fed sub-lethal concentrations of emamectin benzoate at 0.0125, 0.025, 0.05, 0.075, and 0.1 ppm, there was no significant difference in gustatory response between treatments compared with those fed 2.5 M sucrose solutions (F = 0.67; df = 5, 107; p >0.05). In Test 2, emamectin benzoate concentrations was increased to include 0.2, 0.6 and 1.0 ppm to better assess the effects of emamectin benzoate on feeding response and reproduction. Emamectin benzoate did indeed significantly depress gustatory response of females at higher concentrations when compared with those fed 2.5 M sucrose solutions (F = 3.61; df = 5, 84; p < 0.01; Figure 6). *H. zea* females ingested significantly less emamectin benzoate at 1 ppm (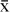 = 72.1 mg/♀), but this value was not significantly different from those which ingested 2.5 M sucrose solution.

### Fecundity

Females deposited significantly fewer eggs at 0.0125, 0.025, 0.05 and 0.1 ppm compared with those fed 2.5 M sucrose solutions in day 1 of Test 1 (Table 2; F = 2.30; df = 5, 22; p <0.10). However, emamectin benzoate did not significantly influence fecundity either in day 2 (F = 1.56; df = 5, 22; p >0.10) or in day 3 of Test 1 (F = 1.24; df = 5, 21; p >0.10). Also, when the total number of eggs deposited during 3 days of testing were pooled, ingestion of emamectin benzoate by female *H. zea* did not influence fecundity (F = 2.05; df = 5, 21; p >0.10).

In contrast with Test 1, emamectin benzoate significantly influenced fecundity of female *H. zea* during all 3 days of testing in Test 2 (Table 3; F = 2.77; df = 5, 20; p <0.05 for day 1; F = 3.19; df = 5, 19; p <0.05 for day 2; and F = 2.71; df = 5, 18; p <0.10 for day 3). There was, however, no consistent trend favoring one treatment of emamectin benzoate over another in reducing fecundity of *H. zea* during three days of testing, probably owing to large variance. When eggs collected over a 3-day period were pooled, the number of eggs deposited by *H. zea* varied significantly between treatments as well (F = 3.05; df = 5, 18; p < 0.05). The number of eggs deposited was the highest at 0.2 ppm with 1240 eggs/♀, but this value was not significantly different from females fed 2.5 M sucrose solutions. In spite of the large variance, a common trend from the data show that emamectin benzoate did reduce oviposition significantly at 0.6 ppm and above.

**Table 3.**
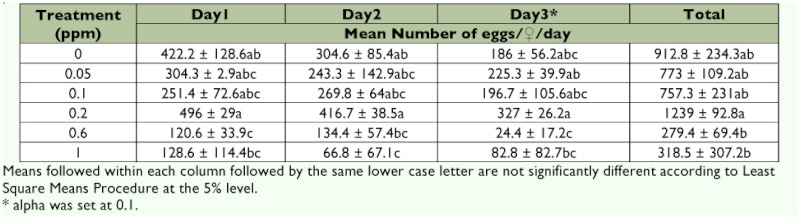
Number of eggs (± SEM) deposited by bollworm fed emamectin benzoate mixed with 2.5 M sucrose during a 3-day period, Test 2.

**Table 4.**
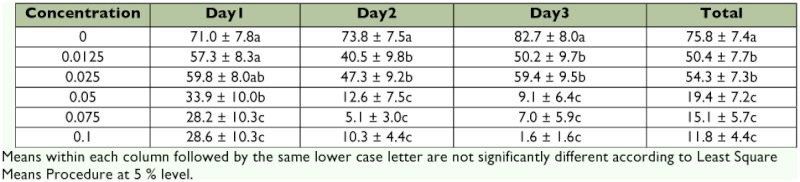
Mean larval hatch (± SEM) of eggs deposited by females fed emamectin benzoate mixed with 2.5 M sucrose, Test 1.

### Fertility

In Test 1, emamectin benzoate significantly depressed percent larval hatch of eggs for all 3 days of testing. The ANOVA statistics for each of these 3 days were: day 1, F = 4.11, df = 5, 61, p <0.005; day 2, F = 13.29, df = 5, 65, p <0.001; and day 3, F = 20.69, df = 5, 61, p <0.001. Table 4 shows that in day 1, emamectin benzoate significantly reduced larval hatch at 0.05 ppm, and in day 2 and day 3, however, significant reduction in larval hatch occurred at a concentration as low as 0.0125 ppm. This suggests a cumulative effect of emamectin benzoate in reducing larval hatch on days 2 and 3 at a lower concentration compared with that on day 1. Emamectin benzoate significantly reduced larval hatch to less than 2% at 0.1 ppm on day 3. Overall trend for all 3 days of observations suggest that percent larval hatch of eggs deposited by females tended to decline significantly with ingestion of increasing concentrations of emamectin benzoate.

Similar to Test 1, emamectin benzoate significantly reduced percent larval hatch of eggs on all 3 days of testing in Test 2 (Table 5). The ANOVA statistics for each of these 3 days were: day 1, F = 8.73; df = 5, 47; p <0.001; day 2, F = 11.05, df = 5, 44, p <0.001; day 3, F = 9.60, df = 5, 40, p <0.001. In contrast to Test 1, reduction in percent larval hatch at 0.05 ppm and 2.5 M sucrose were comparable during all 3 days of testing. This discrepancy in reduction in percent larval hatch at 0.05 ppm between Test 1 and Test 2 probably reflects the difficulty in mixing the insecticide in a viscous medium such as the 2.5 M sucrose solution consistently using the serial dilution technique. Alternatively, it is likely that the use of 1 M sucrose in lieu of 2.5 M sucrose may produce more consistent mixing of test solutions. Reduction in percent larval hatch at 0.1 ppm and above on all three days of testing was more apparent and exhibited a more consistent dose response relationship. Similar to the trend observed in Test 1, reduction in larval hatch appears to be negatively related with ingestion of increasing concentrations of emamectin benzoate by female *H. zea.*

**Table 5.**
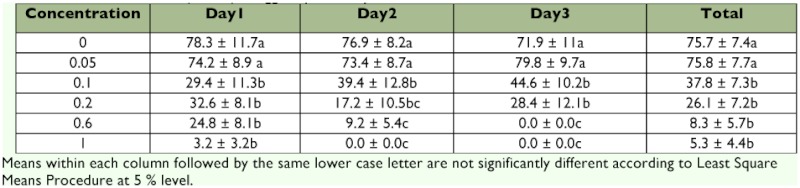
Mean larval hatch (± SEM) of eggs deposited by females fed emamectin benzoate mixed with 2.5 M sucrose, Test 2.

**Figure 7.**
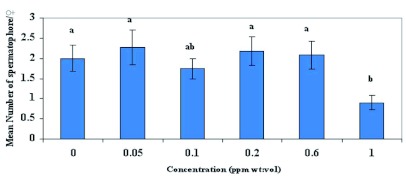
Mean number of *Helicoverpa zea* spermatophores/♀ (± SEM) fed emamectin benzoate mixed with 2.5 M sucrose solution in Test 2. Means followed by the same lower case letter are not significantly different according to Least Square Means Procedure (P=010). High quality figures are available online.

### Spermatophore counts

In Test 1, emamectin benzoate did not significantly influence mating frequency of females compared with those fed 2.5 M sucrose solution (F = 0.55; df = 5, 67; p >0.05). In contrast with Test 1, Figure 7 shows that mating frequency was significantly different between treatments in Test 2 (F = 2.20; df = 5, 55; p <0.10). Mating frequency at 1.0 ppm was significantly less compared with females fed 2.5 M sucrose solutions. There was no significant difference in mating frequency between 0.05, 0.2 and 0.06 ppm and 2.5 M sucrose. Also, mating frequency did not differ significantly between 0.1 and 1.0 ppm. Overall, multiple matings with three to four spermatophores/♀ were common in females that ingested emamectin benzoate. The mean number of spermatophores/♀ in Test 1 and Test 2 were 2.1 and 1.9, respectively. These values were slightly lower than those reported by López *et al.* (1978) and Latheef *et al.* (1991) who reported that natural populations of *H. zea* captured in its primary host, corn, had a mean number of spermatophores of 2.64 and 2.20 per female, respectively.

**Figure 8.**
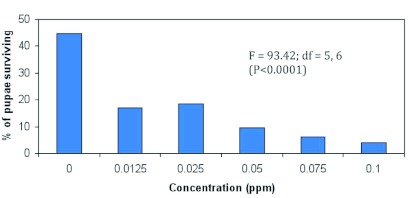
Relationship between larval survival to the pupal stage and concentrationsof emamectin benzoate fed laboratory-reared female *Helicoverpa zea* (Test 1). (Nparl way procedure). High quality figures are available online.

**Figure 9.**
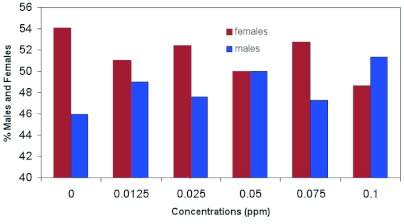
Frequency distribution of female and male *Helicoverpa zea* pupae in Test 1 in which laboratory-reared females were fed emamectin benzoate and paired with untreated males (χ^2^ = 0.98; P > 0.96). High quality figures are available online.

### Survival of pupae

The relationship between percentage of larvae surviving to the pupal stage and emamectin benzoate concentrations is shown in Figure 8.

Emamectin benzoate did account for a significant portion of the variability in larval survival to the pupal stage (Nparlway procedure: F = 93.42; df = 5, 6; p <0.0001; Kruskal-Wallis test: χ^2^ = 10.81; df = 5; p < 9.620E-05). Figure 9 shows that the frequency distribution of female and male pupae was not significantly different between concentrations of emamectin benzoate (χ^2^ = 0.98; df = 5; p >0.96).

## Conclusion

The results reported in this study are in agreement with Argentine *et al.* (2002) who reported using an artificial bioassay to show that emamectin benzoate was most effective in controlling several Lepidoptera including *H. virescens.* Data presented here demonstrate that emamectin benzoate has good potential for use in an attracticide/stimulant formulation for areawide suppression of the *H. zea.* Emamectin benzoate kills adults upon ingestion at very low concentrations, although it is slow-acting. The extremely low amount of emamectin benzoate that suppresses larval hatch of eggs when ingested by laboratory-reared females is a promising characteristic and this coupled with the lack of inhibition of gustatory and proboscis extension at sublethal concentrations makes this chemical an ideal candidate for further research. Furthermore, White *et al.* (1997) reported that emamectin benzoate provides ecological selectivity to a wide range of beneficial arthropods and is compatible with integrated pest management programs. More detailed studies in the laboratory, as well as in the field, are needed to validate the results obtained in this study. Studies are needed to determine the effect of emamectin benzoate on male *H. zea*, relative to fecundity, fertility and mating frequency. Any spray applications against *H. zea* moths must occur during peak emergence of the insect and appropriate application techniques must be developed.

The major advantage of attract-and-kill strategy for *H. zea* is that it is not gender specific, and that feeding habits of both sexes could be exploited for control purpose. Targeting virgin *H. zea* populations before they mate and disperse for reproductive functions is potentially an effective strategy against nocturnal Lepidoptera. The attract-and-kill strategy for *H. zea* appears to be in a conceptual stage and field validation of results reported herein is warranted to move this technology forward.
